# Behavioral effects of disrupted direct pathway signal flow caused by dopamine depletion

**DOI:** 10.1186/1471-2202-14-S1-P205

**Published:** 2013-07-08

**Authors:** Samantha R Summerson, Behnaam Aazhang, Caleb T Kemere

**Affiliations:** 1 Department of Electrical and Computer Engineering, Rice University, Houston, TX 77005, USA; 2 Department of Neuroscience, Baylor College of Medicine, Houston, TX 77030, USA

## 

The basal ganglia (BG) are involved in many functions, including voluntary motor control and cognition, and are the primary nuclei affected by Parkinson's disease (PD). A dominant pathological characteristic of PD is the loss of dopamine-producing neurons in the substantia nigra pars compacta (SNc). Several symptoms of PD, such as increased delays in movement onset and reduced motor ability, can be directly related to the impaired flow of signals through the direct pathway, caused by dopamine depletion in the SNc [1]. This altered computation of neural signaling in the BG affects both motor and associative circuits.

**Figure 1:**
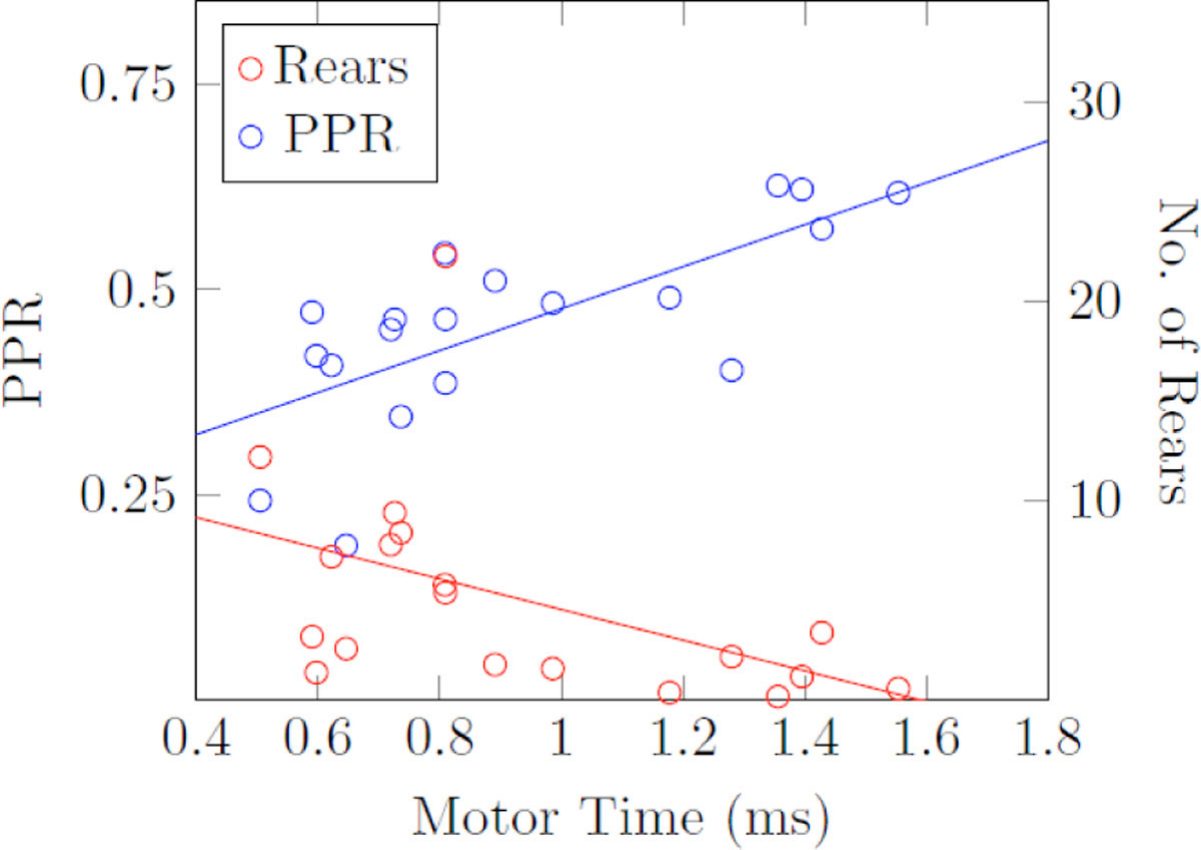
**All data points are from the subjects in both the naïve and hemi-parkinsonian state**. PPR is shown with blue markers and number of rears is shown with red. PPR was significantly correlated with motor time (t-test: p < 0.001). The blue line indicates the linear relationship between the two found via regression. Number of rears was significantly negatively correlated with motor time (t-test: p < 0.05) and the red line is found via linear regression.

We investigated the resultant behavior associated with reduced dopamine levels in the 6-OHDA hemi-parkinsonian rodent model. Postmortem immunohistochemistry revealed a significant mean depletion of 78.95% of dopaminergic cells in the SNc. The subjects (n = 10) were trained in a reaction time task, which was used to quantify reaction time (i.e. time to movement onset), motor time (i.e. time to complete motor action), and the proportion of premature responses (PPR) to behavior cues, which is a measure impulsive behavior [2]. The level of apathy was measured as the number of rears in an open field environment [3]. The performance of all subjects was quantified in the naïve state and hemi-parkinsonian state. Between the two states there was a significant difference for all variables considered. Additionally we found strong links between the motor and cognitive behaviors. Motor time significantly increased in the hemi-parkinsonian state and was positively correlated with impulsivity. Motor time was simultaneously negatively correlated with the number of rears in a 5-min epoch. These conclusions are supported by Figure 1. Thus, loss of dopamine simultaneously modulates motor performance, impulsivity and apathy. Our results reveal how dopamine tuning the excitability of the direct pathway has coupled motor and cognitive performance effects. This that demonstrates the role dopamine plays in information processing and computation within the motor and associative circuits should not be considered independently.
